# Organ-specific responses during brain death: increased aerobic metabolism in the liver and anaerobic metabolism with decreased perfusion in the kidneys

**DOI:** 10.1038/s41598-018-22689-9

**Published:** 2018-03-13

**Authors:** A. C. Van Erp, R. A. Rebolledo, D. Hoeksma, N. R. Jespersen, P. J. Ottens, R. Nørregaard, M. Pedersen, C. Laustsen, J. G. M. Burgerhof, J. C. Wolters, J. Ciapaite, H. E. Bøtker, H. G. D. Leuvenink, B. Jespersen

**Affiliations:** 1 Department of Surgery, University Medical Center Groningen, Groningen, The Netherlands; 2 Institute for Medical and Biological Engineering, Schools of Engineering, Biological Sciences and Medicine, Pontificia Universidad Católica de Chile, Santiago, Chile; 3 Department of Cardiology, Aarhus University Hospital, Aarhus, Denmark; 4 Department of Clinical Medicine, Aarhus University, Aarhus, Denmark; 5 MR Research Center, Clinical Institute, Aarhus University, Aarhus, Denmark; 6 Department of Epidemiology, University Medical Center Groningen, Groningen, The Netherlands; 7 Department of Analytical Biochemistry, Research Institute of Pharmacy, University of Groningen, Groningen, The Netherlands; 8 Systems Biology Centre for Energy Metabolism and Ageing, University of Groningen, Groningen, The Netherlands; 9 Department of Paediatrics, University Medical Center Groningen, Groningen, The Netherlands; 10 Department of Renal Medicine, Aarhus University Hospital, Aarhus, Denmark

## Abstract

Hepatic and renal energy status prior to transplantation correlates with graft survival. However, effects of brain death (BD) on organ-specific energy status are largely unknown. We studied metabolism, perfusion, oxygen consumption, and mitochondrial function in the liver and kidneys following BD. BD was induced in mechanically-ventilated rats, inflating an epidurally-placed Fogarty-catheter, with sham-operated rats as controls. A 9.4T-preclinical MRI system measured hourly oxygen availability (BOLD-related R2*) and perfusion (T1-weighted). After 4 hrs, tissue was collected, mitochondria isolated and assessed with high-resolution respirometry. Quantitative proteomics, qPCR, and biochemistry was performed on stored tissue/plasma. Following BD, the liver increased glycolytic gene expression (*Pfk-1*) with decreased glycogen stores, while the kidneys increased anaerobic- (*Ldha*) and decreased gluconeogenic-related gene expression (*Pck-1*). Hepatic oxygen consumption increased, while renal perfusion decreased. ATP levels dropped in both organs while mitochondrial respiration and complex I/ATP synthase activity were unaffected. In conclusion, the liver responds to increased metabolic demands during BD, enhancing aerobic metabolism with functional mitochondria. The kidneys shift towards anaerobic energy production while renal perfusion decreases. Our findings highlight the need for an organ-specific approach to assess and optimise graft quality prior to transplantation, to optimise hepatic metabolic conditions and improve renal perfusion while supporting cellular detoxification.

## Introduction

The shortage of donor organs suitable for transplantation remains a major healthcare challenge. Despite various strategies to expand the donor pool, such as the increased use of non-heart beating, or living donor grafts^1,2^, most organs transplanted worldwide are still obtained from brain-dead donors^3^. However, compared to living donor transplantation, transplantation of brain-dead organ grafts leads to higher rejection rates and inferior long-term outcomes^4–6^. Thus, the current challenge is to use all available organs including the suboptimal and concurrently improve transplantation outcomes.

Brain death (BD) causes complex disturbances in body homeostasis^4,6–8^. BD is the result of increased intracranial pressure (ICP), which leads to progressive ischemia of the cerebrum, brain stem, and spinal cord. Consequently, this triggers a sympathetic response with catecholamine release, which causes systemic vasoconstriction and decreased flow through peripheral organs including the liver and kidneys^9–11^. Furthermore, impairment of the hypothalamus and pituitary gland results in hormonal disturbances, including reduced levels of circulating triiodothyronine, vasopressin, and cortisol^10^. Eventually, ischemia of the spinal cord results in the loss of sympathetic tone in the peripheral vascular bed, potentially impacting future organ grafts^11,12^. Regarding the liver and kidneys, this results in increased injury biomarkers pertaining to apoptosis, immune activation, inflammation, and oxidative stress^4,6–8,13^. Treatments administered to brain-dead donors aimed at improvement of post-transplantation graft and recipient survival may be of benefit. However, systematic reviews show no consistent evidence for the effectiveness of any such treatments^14,15^. Hence, new strategies are needed to assess and optimise organ quality prior to transplantation.

We initiated the present study to investigate metabolic alterations during BD in the liver and kidneys. Under normal circumstances, both organs are metabolically active, i.e. the liver primarily fulfils a synthesis and detoxification function and the kidneys an excretory task. The liver is considered the predominant site for carbohydrate, lipid, and amino acid metabolism^16^. Nevertheless, the kidneys are responsible for supplying up to half of the total blood glucose levels during prolonged fasting or starvation^16,17^. These metabolic processes are tightly regulated as perturbances in cellular metabolic checkpoints e.g. changes in nutrient or oxygen supply can initiate both apoptosis and necrosis^18^. Considering the neuro-endocrine dysregulation, as well as haemodynamic impairment and inflammation during BD, it is conceivable that metabolism is altered during BD and that this influences the quality of transplantable organs.

Few studies have explored metabolic changes in the brain-dead donor. Novitzky *et al*. reported decreased metabolite utilization and accumulation of fatty acids and lactate in plasma, suggesting a shift from aerobic to anaerobic metabolism^19^. Similar results in the myocardium of brain-dead pigs point towards increased anaerobic metabolism in combination with decreased ATP levels^20^. These studies suggest that the observed metabolic changes are caused either by impaired oxygen utilization due to a primary metabolic (i.e. mitochondrial) impairment or alternatively, by impaired oxygen delivery due to changes in tissue perfusion^21^. Mitochondrial impairment can cause increased anaerobic ATP production as well as oxidative stress^22,23^ and has previously been observed in the hearts of brain-dead pigs^24^ and the muscle fibres of brain-dead, human subjects^21^. A previous study by our group showed decreased ATP levels in the kidney following BD when compared to living donation^24^. However, mitochondrial function following BD in the liver and kidneys has not been examined. Alternatively, anaerobic alterations could result from changes in peripheral perfusion in the hemodynamically unstable brain-dead donor. Animal studies have shown decreased perfusion of the liver and kidneys immediately following the sympathetic storm during BD^25^ as well as after traumatic brain injury^26^. However, perfusion of the liver and kidneys during BD has not yet been explored. Interestingly, a recent publication shows that mitochondrial and metabolic pathways are most pre-dominantly affected following BD^27^. Together, this suggests that exploration of metabolic changes during BD and possible underlying causes, will provide insights into organ-specific metabolic alterations prior to preservation and might justify (pre)conditioning of grafts prior to transplantation.

The purpose of this study was to investigate the influence of BD on systemic and specifically hepatic and renal metabolism in a rodent BD model. We hypothesised that previously observed anaerobic changes during BD originated at least in part from either mitochondrial dysfunction or impaired peripheral perfusion in the liver and kidneys. To test this hypothesis, we used repetitive *in vivo* magnetic resonance imaging (MRI) to visualise tissue perfusion and oxygenation. Furthermore, we assessed mitochondrial function by measuring *in vitro* mitochondrial respiration as well as mitochondrial proteomics after 4 hrs of BD.

## Results

### Brain death parameters

Adult, male rats were randomly assigned to the BD (n = 8) or sham-operated (sham) group (n = 8). Intracranial pressure (ICP), mean arterial pressure (MAP), and oxygen saturation were measured continuously throughout the experiment. As an internal control for the BD model, declaration of BD was confirmed when the ICP superseded the MAP and consequently cerebral perfusion pressure (CPP) was lower than 0 mmHg (Fig. S1A). Induction of BD showed a uniform MAP pattern consistent with previous studies^8,28^, with a mean time of 29.3 ± 6.0 min to declare BD (Fig. S1B). The MAP of all animals was maintained above 80 mmHg throughout the experiment without the use of vasopressors or colloids. One out of eight experimental brain-dead animals had a CCP higher than 0 mmHg due to an obstruction of the ICP catheter, but as it showed a characteristic MAP profile and absent corneal and pupillary reflexes, the animal was included in the study (Fig. S1).

### Plasma and tissue functional and injury markers, metabolites, and pH following brain death

**Figure 1:**
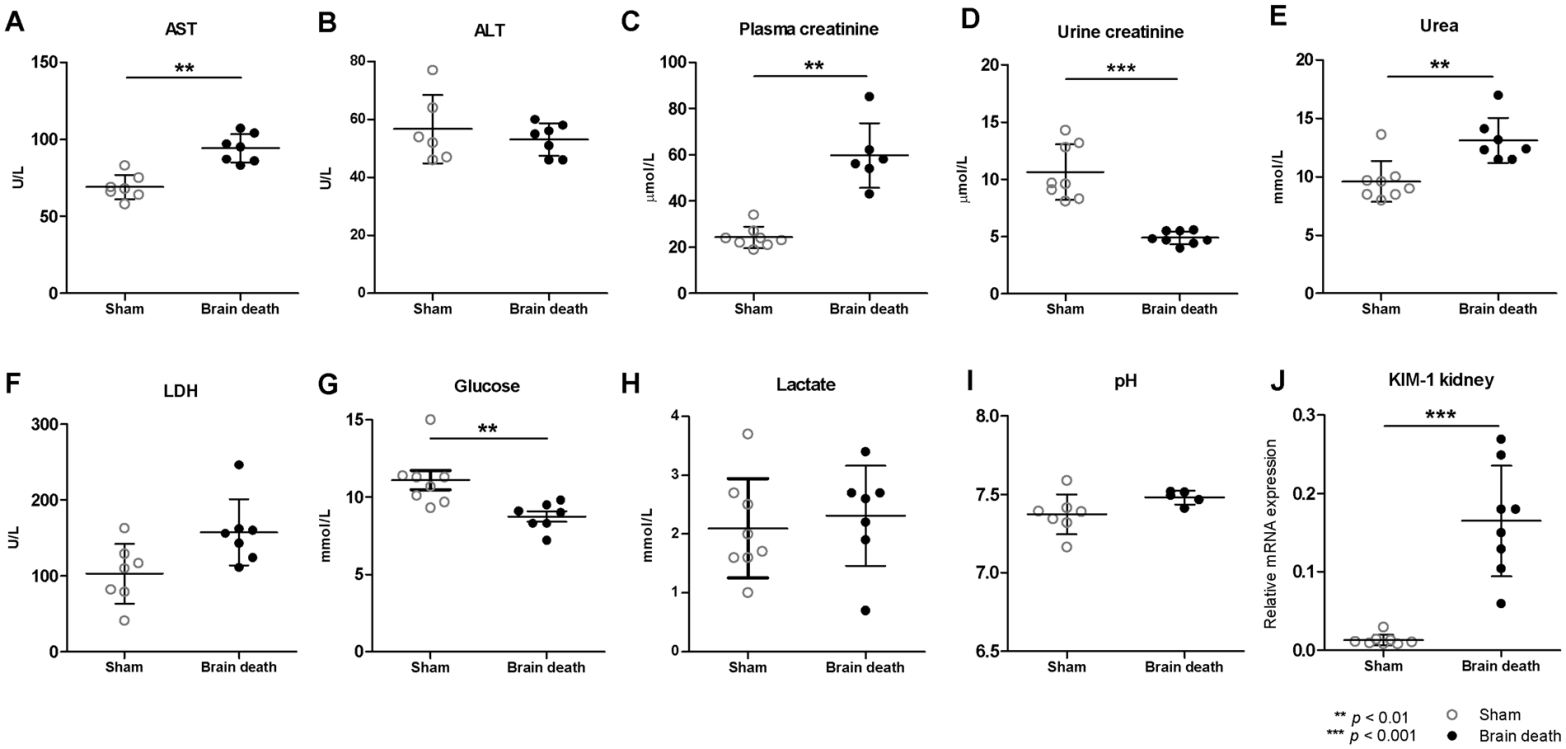
Brain death induced renal failure and caused increased AST and lactate levels, yet decreased glucose levels in plasma. (**A**) Aspartate transaminase, (**B**) alanine transaminase, (**C**) plasma creatinine, (**D**) urine creatinine (**E**) urea, (**F**) lactate dehydrogenase, (**G**) glucose, and (**H**) lactate levels in plasma; (**I**) pH determined with blood gas analyses; and (**J**) relative gene expression of Kidney Injury Molecule-1 *(Kim-1)* after 4 hrs of experimental time. Results are presented as mean ± SD, n = 7 per group (**p < 0.01).

We performed plasma, urine, and blood gas analyses of several injury markers and plasma metabolites to validate our model and to ensure that our results were in line with previous human and animal studies, showing the negative impact of BD on renal and hepatic function as well as carbohydrate and fatty acid metabolism. Furthermore, we assessed renal mRNA expression of kidney injury molecule-1 (KIM-1), which is an early biomarker for BD-induced injury in the kidney prior to transplantation^29^. In plasma of brain-dead animals, increased levels of hepatic injury marker aspartate transaminase (AST, 94.14 ± 9.25 vs. 69.0 ± 8.04, *p* = 0.001) but not alanine transaminase (ALT, 53.14 ± 5.61 vs. 56.67 ± 11.86 vs. *p* = 0.829) was found compared to sham animals (Fig. 1A,B). Renal functional markers urea (13.14 ± 1.94 vs. 9.61 ± 1.75, *p* = 0.006) and plasma creatinine (59.67 ± 13.95 vs. 24.25 ± 4.59, *p* = 0.001) were also increased following BD (Fig. 1C,E), whereas urine creatinine was decreased (4.90 ± 0.58 vs. 10.64 ± 2.42, *p* < 0.001, Fig. 1D). Lactate dehydrogenase (LDH) levels were higher in the brain-dead group (157.4 ± 43.50 vs. 103.0 ± 39.54, *p* = 0.053, Fig. 1F). mRNA expression of *Kim-1* was increased in the kidney of brain-dead versus sham animals (0.165 ± 0.071 vs. 0.013 ± 0.007, *p* < 0.001, Fig. 1J).

Plasma glucose concentrations were significantly reduced following BD (8.74 ± 0.88 vs. 11.10 ± 1.76, *p* = 0.005, Fig. 1G), yet lactate levels (2.31 ± 0.85 vs. 2.10 ± 0.84, *p* = 0.450, Fig. 1H) and pH (7.48 ± 0.04 vs. 7.37 ± 0.13, *p* = 0.073, Fig. 1I) were not different between the two groups. Results from one sham animal indicated supraphysiological values of these plasma markers, which was confirmed statistically with an outlier test. As a result, this animal was removed from the analyses.

**Figure 2:**
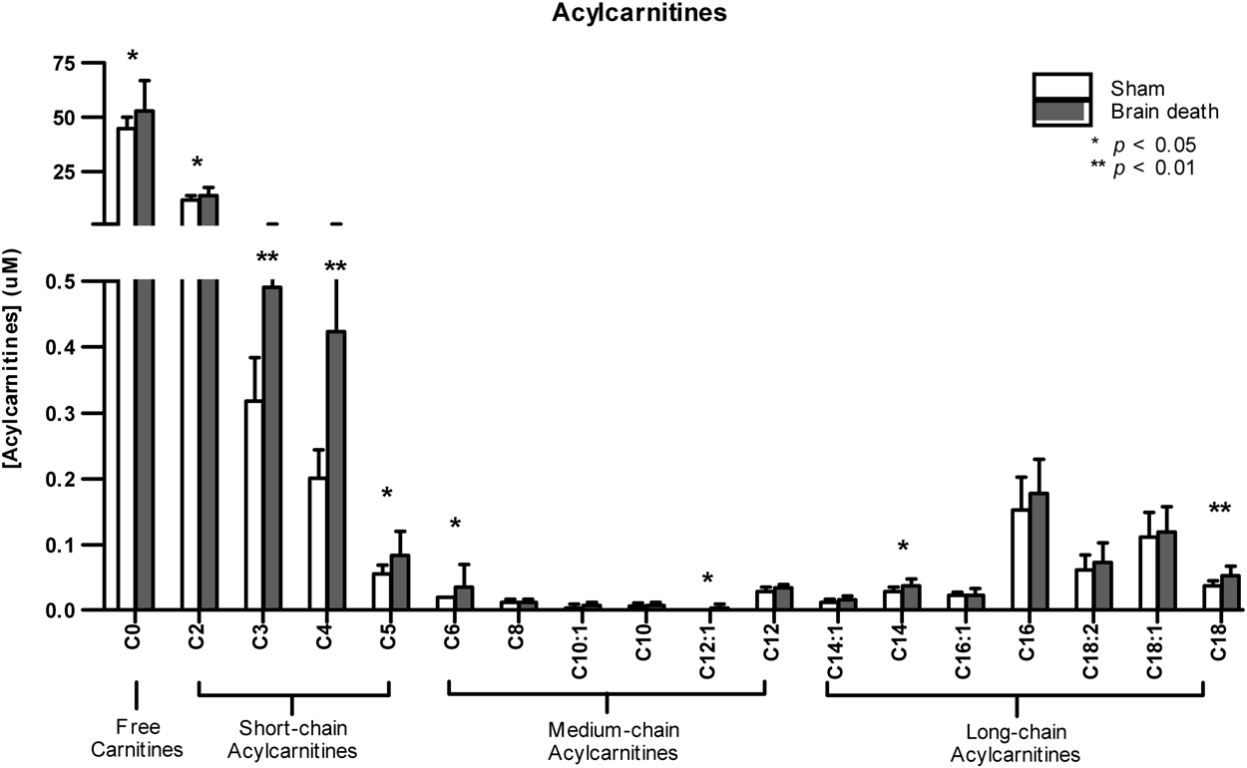
Brain death increased fatty acid β oxidation. After 4 hrs of experimental time, plasma concentrations of saturated and unsaturated acylcarnitines were measured in sham and brain-dead rats with different carbon (C) chain lengths: short (C0–C5), medium (C6–C12), and long (C14–C18) chain acylcarnitines. Data are represented as mean ± SD, n = 8 per group (*p < 0.05, **p < 0.01, compared to sham).

Several types of fatty acid metabolites were assessed to determine how BD affects fatty acid oxidation (FAO) and specifically which types of fatty acids were altered. Firstly, concentrations of free carnitine (C0), which serves as a shuttle for long-chain (C14–C18) acylcarnitines (LCAC) into the mitochondria, were higher in brain-dead versus sham animals (C0: 56.50 ± 10.20 vs. 44.75 ± 5.23, *p* < 0.05). LCAC concentrations were slightly elevated in the plasma of brain-dead animals (C14: 0.04 ± 0.01 vs. 0.03 ± 0.01, all *p* < 0.05; C18: 0.06 ± 0.01 vs. 0.04 ± 0.01, *p* < 0.05, Fig. 2). Short-chain (C3–C4) acylcarnitines (SCAC) and medium-chain (C6–C12) acylcarnitines (MCAC) can permeate the mitochondrial membrane without a carnitine shuttle and serve as additional fuel for FAO and the TCA cycle. SCAC concentrations were significantly increased in the plasma of brain-dead animals (C2: 14.52 ± 2.93 vs. 11.70 ± 2.14, C5: 0.09 ± 0.03 vs. 0.06 ± 0.01, both *p* < 0.05; C3: 0.50 ± 0.16 vs. 0.32 ± 0.07, C4: 0.20 ± 0.04 vs. 0.18 ± 0.04, both *p* < 0.01, Fig. 2), whereas minimal changes in MCAC concentrations were observed (C6: 0.04 ± 0.03 vs. 0.02 ± 0.0, C12:1: 0.01 ± 0.01 vs. 0.00 ± 0.00, Fig. 2).

### Glucose metabolism and glycogen storage in the liver and kidneys following brain death

**Figure 3:**
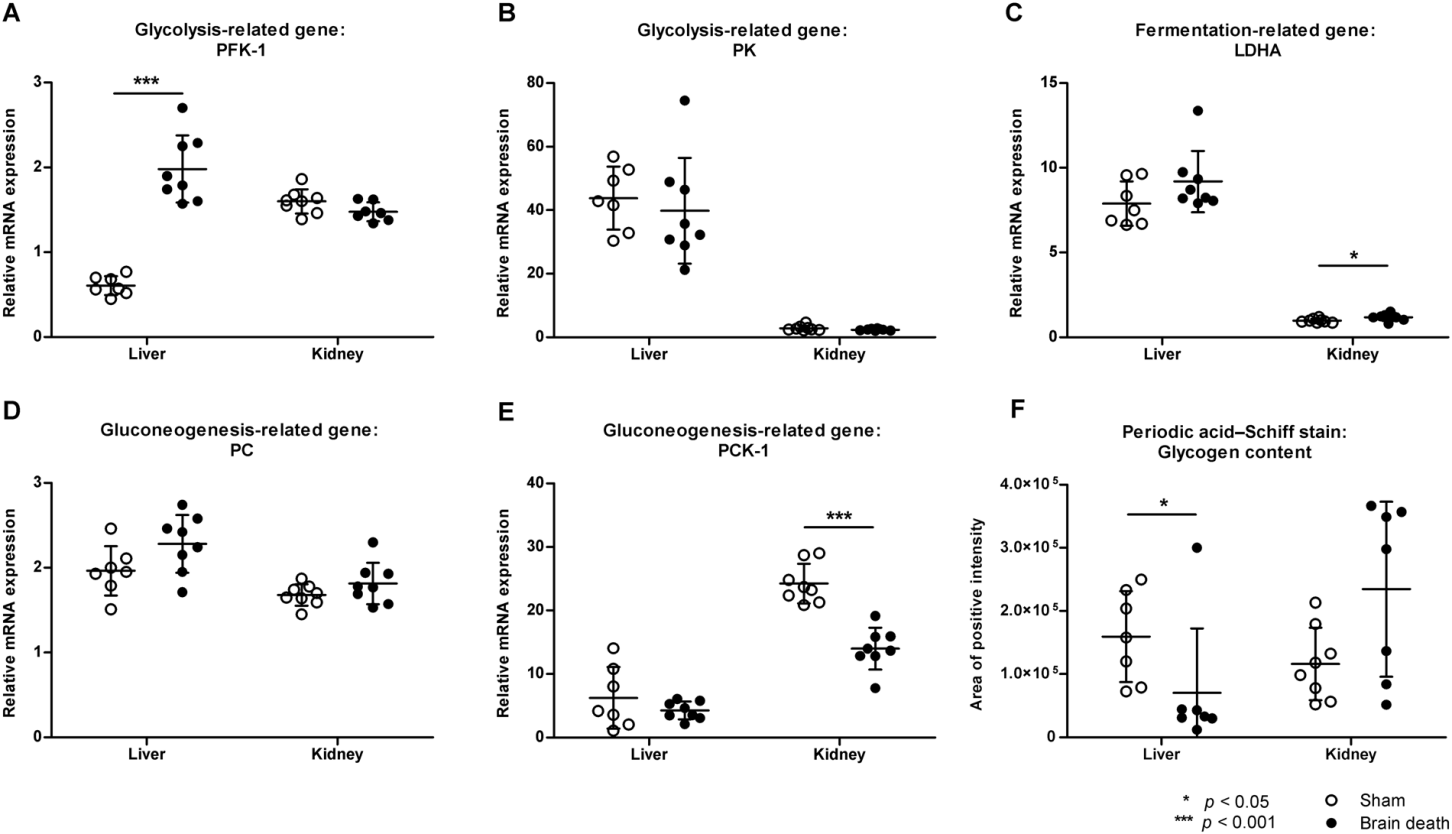
Carbohydrate metabolism-related gene expression profiles and glycogen content in the liver and kidney after 4 hrs of brain death. Relative gene expression of glycolysis related genes (**A**) Phosphofructokinase-1 (*Pfk-1*) and (**B**) Pyruvate kinase (*Pk*), (**C**) anaerobic glycolysis-related gene Lactate dehydrogenase A (*Ldha*), and gluconeogenesis related genes (**D**) Pyruvate carboxylase (*Pc*) and (**E**) PEP carboxykinase 1 (*Pck-1*). (**F**) Periodic Acid–Schiff staining of glycogen, overall quantification at 20 × magnification. Results are presented as mean ± SD, n = 8 per group (*p < 0.05, ***p < 0.001).

We assessed mRNA expression of several glycolytic and gluconeogenic enzymes to evaluate hepatic and renal glucose metabolism. Expression of the glycolytic enzyme phosphofructokinase-1 (*Pfk-1*) was increased in the liver (1.98 ± 0.40 vs. 0.61 ± 0.11, *p* < 0.001), but not the kidney (1.48 ± 0.11 vs. 1.60 ± 0.14, *p* = 0.319) of brain-dead versus sham animals (Fig. 3A). mRNA levels of glycolytic enzyme pyruvate kinase (*Pk*) did not differ between groups in the liver (39.8 ± 17.0 vs. 43.8 ± 9.89, *p* = 0.336) and kidney (2.39 ± 0.30 vs. 2.86 ± 0.77, *p* = 0.130) (Fig. 3B). The gluconeogenic enzyme pyruvate carboxylase (*Pc*) was similarly expressed in both groups in the liver (2.28 ± 0.34 vs. 1.96 ± 0.29, *p* = 0.093) and kidney (1.81 ± 0.25 vs. 1.68 ± 0.13, *p* = 0.293), whereas PEP carboxykinase-1 (*Pck-1*) expression was significantly lower in the kidneys of brain-dead than in sham animals (14.0 ± 3.28 vs. 24.2 ± 3.13, *p* < 0.001) yet not different between groups in the liver (4.23 ± 1.41 vs. 6.26 ± 4.83, p = 0.694, Fig. 3E). Expression of the fermentation-related enzyme lactate dehydrogenase A (*Ldha*) was not different between groups in the liver (9.19 ± 1.81 vs. 7.89 ± 1.31, *p* = 0.190), whereas increased expression was observed in the kidney of brain-dead versus sham groups (1.19 ± 0.21 vs. 0.98 ± 0.12, *p* = 0.038) (Fig. 3C). Liver glycogen levels estimated with Periodic Acid–Schiff (PAS) staining showed a decrease in positively stained areas in the liver (7.04 × 10^4^ ± 1.02 × 10^5^ vs. 1.60 × 10^5^ ± 7.20 × 10^4^, *p* = 0.026), but not the kidney (2.35 × 10^5^ ± 1.39 × 10^4^ vs. 1.16 × 10^5^ ± 5.75 × 10^4^ *p* = 0.151) of brain-dead versus sham animals (Figs 3F, S2).

### No changes in mitochondrial respiration in the liver and kidneys following brain death

**Figure 4:**
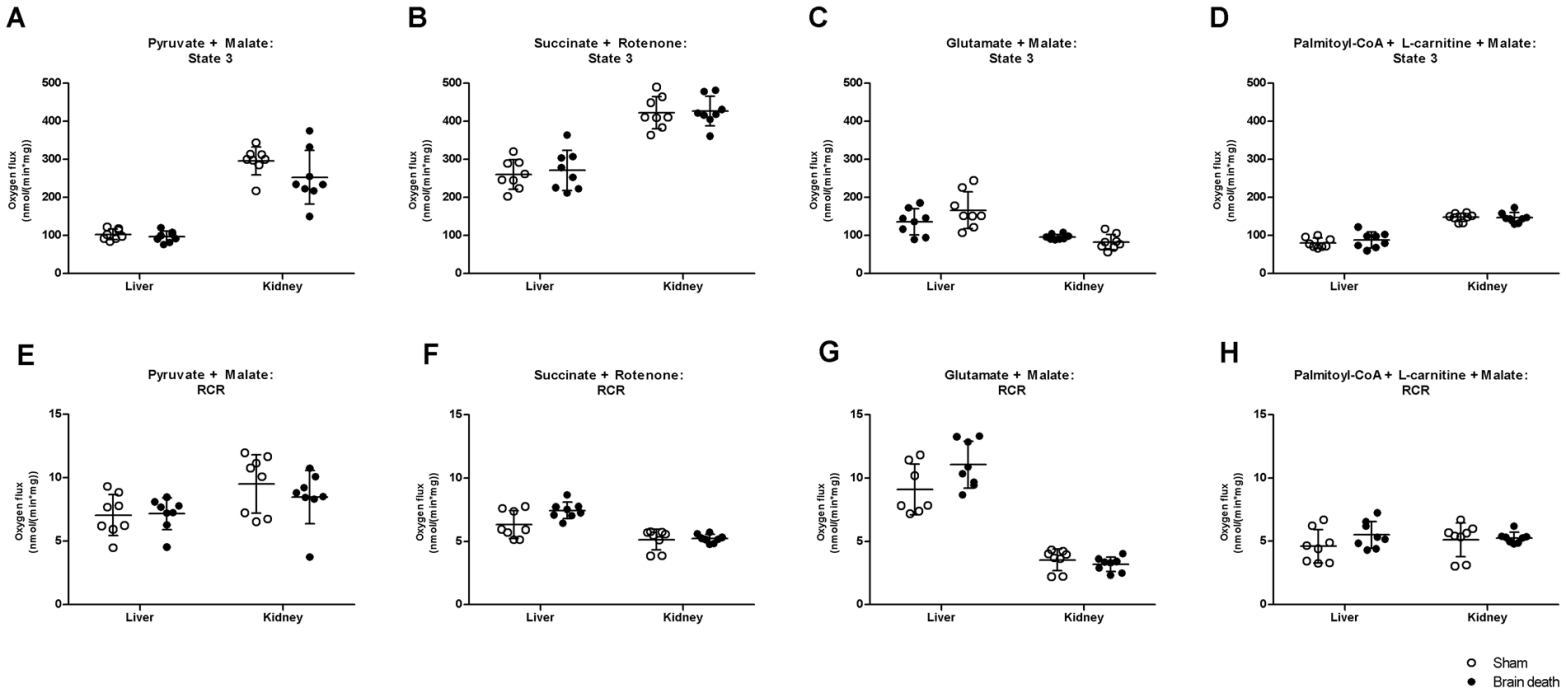
Mitochondrial respiration is unaffected in the liver and kidney following brain death. Maximal ADP-stimulated (state 3) O_2_ consumption rate and Respiratory Control Ratio (RCR) tested using substrates related to (**A**,**E)** the TCA cycle (Glutamate + Malate); (**B**,**F**) complex II-dependent respiration (Succinate + Rotenone); (**C**,**G**) glutamate transaminase (Glutamate + Malate); and (**D**,**H**) fatty acid β-oxidation (Palmitoyl-CoA + L-carnitine + Malate). Results are presented as mean ± SD, n = 8 per group.

Immediately after organ retrieval, mitochondrial function was assessed in isolated hepatic and renal mitochondria by measuring O_2_ consumption rates using four different substrate combinations in different metabolic states (TCA cycle, glutamate transaminase, complex II-dependent respiration, and FAO) (Fig. 4A–D). We found no significant changes in the maximal ADP-stimulated O_2_ consumption rate (state 3) in either the liver or kidney, indicating that the capacity to produce ATP through the oxidative phosphorylation pathway was not affected by BD. Mitochondrial quality control was assessed with the respiratory control ratio (RCR), to detect any changes in oxidative phosphorylation capacity related to tightness of mitochondrial coupling. The RCRs were not significantly different in brain-dead and sham animals when tested with any of the four substrate combinations (Fig. 4E–H).

### Increased hepatic oxygen consumption (BOLD) and decreased renal perfusion (ASL) following brain death

**Figure 5:**
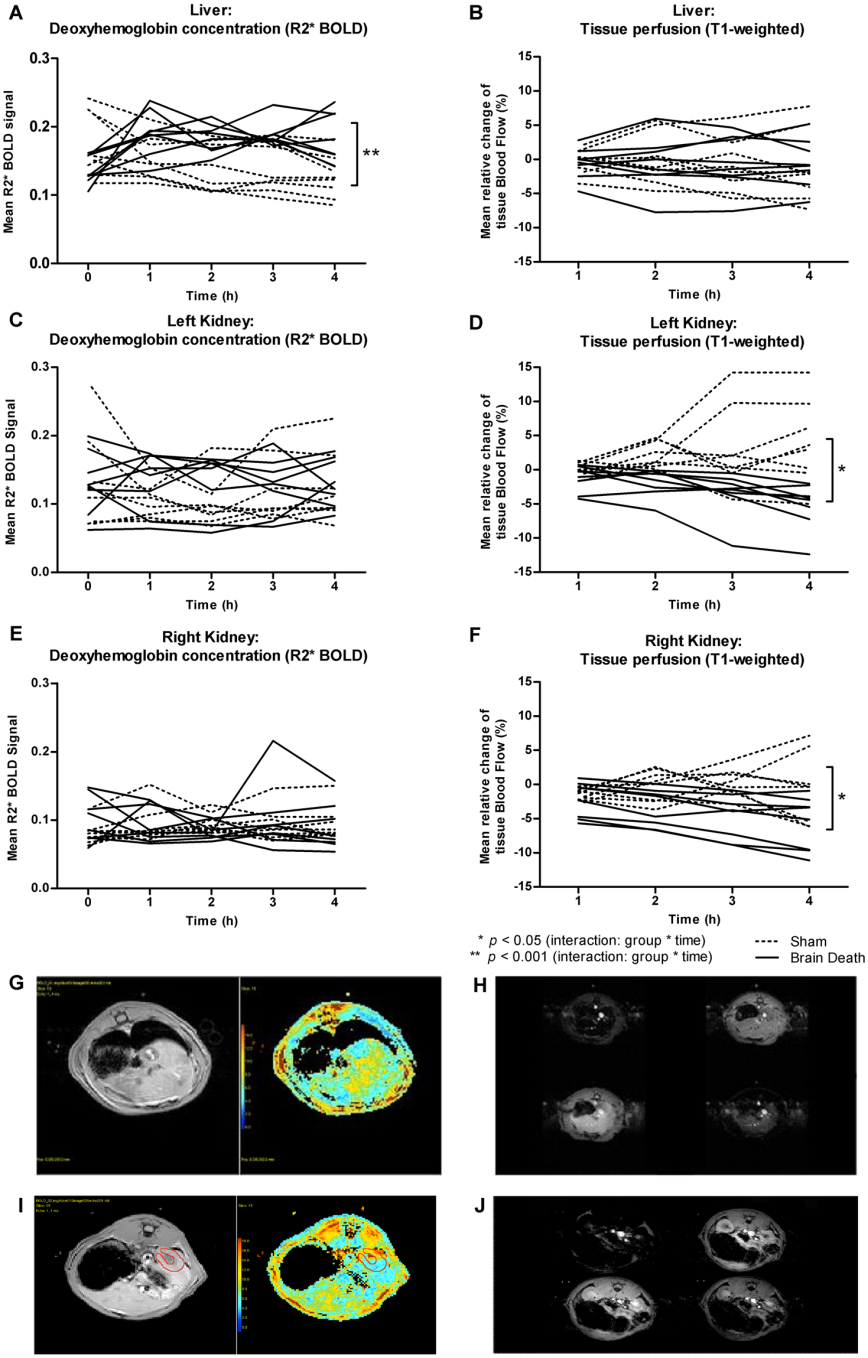
Increased hepatic deoxyhaemoglobin concentration and decreased renal blood flow during brain death. (**A**,**C**,**E**) Hourly, R2* BOLD Magnetic Resonance Imaging (MRI) was performed to estimate deoxyhaemoglobin levels in brain-dead and sham rats, where time “0” represents baseline measurements. (**B,D,F**) Hourly, T1-weighted MRI data was used to estimate the relative change in tissue blood flow compared to baseline measurements in brain-dead and sham animals. Results are presented as mean ± SD, n = 8 per group (interaction group * time: *p < 0.05, **p < 0.001) (**G**,**I**) An example of a greyscale T2 map for liver (**G**) and kidney (**I**) tissue with on the left-hand side a grayscale, T2 signal image. On the right-hand side a T2*-weighted signal is represented as a colour map. (**H,J**) Examples of T1 signal images of liver (**H**) and kidney (**J**) tissue at different inversion times. From the top left to bottom right the signal passes from the vessels through to the tissue.

Throughout the 4 hr BD period, hourly MRI data was obtained to determine Blood Oxygen Level Dependent (BOLD)-related oxygenation and Arterial Spin Labelling (ASL)-related perfusion of the liver and kidneys. BOLD MRI relies on differences in oxygenated and deoxygenated haemoglobin concentrations in blood vessels and surrounding tissue, which in turn causes contrast in the spin-spin relaxation rate (R2*)^30,31^. Differences in the BOLD-related R2* signal can then be correlated to oxygen availability in the tissue of interest, where low R2* values indicate low oxygen consumption yet high oxygenation, and vice versa. R2* baseline values were not different between sham and brain-dead animals in the liver and kidneys (Fig. 5A,C,E). We used a linear mixed model to determine whether MRI signals were significantly different between groups over time. For R2* BOLD values in the liver, we found a significant interaction between time and treatment group (*p* < 0.001), indicating a significant increase in oxygen consumption in brain-dead versus sham animals over time (Fig. 5A). Estimated effects in the liver were: BOLD BD = 0.157 + 0.009 * time; BOLD sham = 0.168–0.011 * time. In the kidneys, there was no significant effect of treatment and time nor a significant interaction effect between groups (Fig. 5C,E).

Arterial spin labelling (ASL) MRI relies on a difference in the T1-weighted signal of inflowing blood compared to that of the tissue of interest. This signal difference can be used to estimate relative changes in tissue perfusion and microcirculation^32^. In the liver, we found no significant effect of treatment and time nor any significant interaction effect between groups (Fig. 5B). In contrast, in each of the kidneys, the linear mixed model for relative T1-weighted perfusion found significant interactions between time and treatment group (*p* < 0.05) in both kidneys (Fig. 5D,F). These significant differences in ASL signal between the two treatment groups over time indicate a decline in perfusion of the kidneys throughout the course of BD. Estimated effects for the left kidney were: ASL BD = 0.429–1.356 * time; ASL sham = −0.638 + 1.193 * time. Estimated effects for the right kidney were: ASL BD = −1.113–1.160 * time; ASL sham = −0.524 + 0.305 * time.

### Increased metabolism-related protein expression in the liver and decreased expression in the kidney

**Figure 6:**
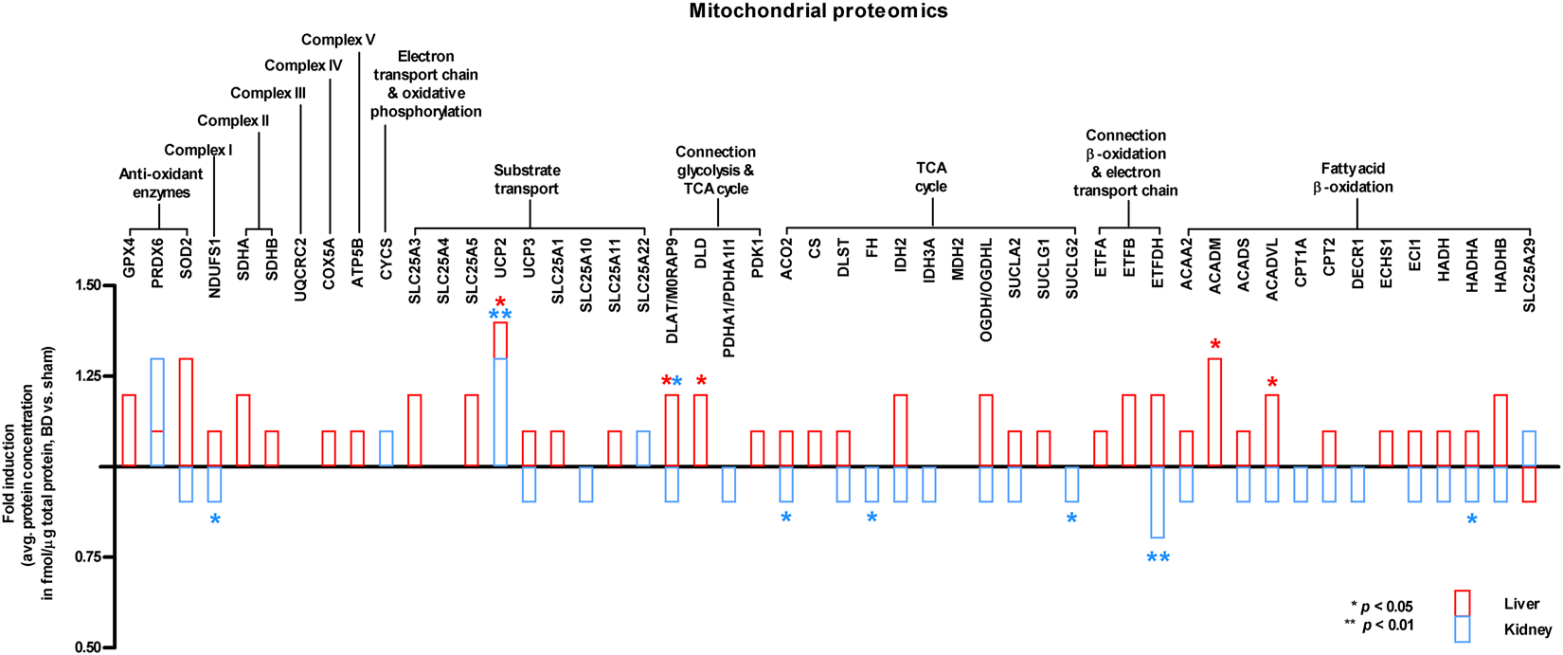
Mitochondrial proteomics profile. Data are represented as mean fold induction of average protein concentration (fmol/µg total protein) in BD versus sham groups in the liver and kidney. Differences in protein concentrations are considered significant when *p* < 0.05 in BD versus sham groups per individual organ, n = 8 per group.

Using a targeted, quantitative proteomics approach, we quantified 50 proteins involved in oxidative phosphorylation, TCA, FAO, and substrate transport, as well as several antioxidant enzymes, in isolated hepatic and renal mitochondria. In the liver of brain-dead animals, we observed increased concentrations of peptides involved in substrate transport (*Ucp2*), the connection between glycolysis and TCA cycle (*Dld* and *Dlat*), and FAO (*Acadm* and *Acadvl*) (*p* < 0.05, Fig. 6). Interestingly, most significant changes in the kidney showed decreased peptide concentrations. These proteins were related to complex I (*Ndufs1*), TCA cycle (*Aco2*, *Fh*, and *Suclg2*) and the connection between FAO and electron transport chain (*Etfdh*), and FAO (*Hadhb*) (*p* < 0.05, Fig. 6). The expression of two renal proteins, involved in substrate transport (*Ucp2*) and the TCA cycle (*Dlat*), was significantly higher in brain-dead compared to sham animals (*p* < 0.05, Fig. 6).

To estimate how these changes in protein expression could influence the metabolic status of the potential grafts, cellular ATP content was measured. We showed reduced ATP levels in both the liver (35.56 ± 15.7 vs. 66.2 ± 19.0) and kidney (8.54 ± 3.13 ± 18.5 ± 3.87) of brain-dead animals (both *p* < 0.001, Fig. S3A), suggesting a decreased bio-energetic efficiency of both organs following BD.

The significantly different renal expression of a peptide belonging to complex I, as well as reduced ATP levels in both liver and kidney, led us to investigate the activities of complex I and ATP synthase individually. We observed no differences in complex I and ATP synthase activity comparing brain-dead versus sham animals (Fig. S3B,C).

### Hepatic and renal oxidative stress markers

Tissue oxidative stress markers were assessed to evaluate how the observed changes in perfusion and oxygenation affected the liver and kidneys. Malondialdehyde (MDA), which relates to the amount of lipid peroxidation by reactive oxygen species (ROS), was increased in the kidney (2.78 ± 0.62 vs. 1.83 ± 0.35, *p* = 0.003), but not the liver (1.87 ± 0.57, *p* = 0.442), of brain-dead compared to sham animals (Fig. S4A). Furthermore, expression of the stress-response protein Heme oxygenase-1 (*Ho-*1) was increased in the liver (1.15 ± 0.34 vs. 0.68 ± 0.23, *p* = 0.017), while no significant changes were observed in the kidney (0.76 ± 0.90 vs. 0.31 ± 0.50, *p* = 0.068) in brain-dead versus sham animals (Fig. S4B).

## Discussion

The energy status of brain-dead donors is a determining factor in renal and hepatic graft survival. In cadaveric donors, a positive correlation exists between pre-transplantation renal high-energy phosphates and post-transplantation graft viability^33,34^. Furthermore, delayed graft function (DGF) in renal transplantation is preceded by a failure to recover aerobic respiration^35^. In the liver of brain-dead donors, blood ketone body ratio (KBR), reflective of hepatic energy production^36,37^, correlates with post-transplant graft function^38^. Furthermore, the ability to increase and maintain KBR was a better predictor of graft survival than conventional liver function tests^39^. These studies strongly suggest that pre-transplant energy status of the liver and kidney is tightly linked to post-transplant graft survival and positive transplantation outcomes. Importantly, our data shows that the energetic and metabolic status of both the liver and kidney are affected already before organ procurement, that is within the brain-dead donor.

This study indicates that the liver increases metabolic activity to meet the metabolic demands imposed by BD pathophysiology. We not only observed lower levels of carbohydrate metabolites, but also increased expression of *Pfk1*, the key enzyme regulating the flux through glycolysis, and increased oxygen consumption as suggested by BOLD-MRI, together indicative of increased aerobic glycolysis. Furthermore, these changes appear to be related to increased glucose catabolism in response to higher energy demands and not the result of impaired cellular respiration (i.e. mitochondrial dysfunction) or perfusion. The altered glucose metabolism is most probably a direct consequence of the catecholamine storm elicited by BD, as catecholamines stimulate aerobic glycolysis and glucose release via either glycogenolysis or gluconeogenesis^40^. Additionally, ATP depletion can result in *Pfk1* activation, producing TCA cycle-substrates such as pyruvate^16^. As glycolysis and gluconeogenesis are reciprocally regulated, it is not surprising that we did not observe any changes in gluconeogenic enzyme expression^1,2,16^. Altogether, our findings seem to reflect increased metabolic utilization of carbohydrate energy sources in the liver because of increased energy demands.

When carbohydrate levels are low, as is seen during periods of fasting and starvation, metabolic pathways shift to facilitate catabolism of fatty acids or amino acids^3,16,17,41^. In line with prior studies in brain-dead patients^4–6,10,19^, we indeed observed increased plasma levels of free fatty acids and amino acids following BD. More specifically, both free carnitine (C0) and several LCAC were increased following BD, suggestive of increased FAO as C0 shuttles LCAC to the mitochondria. Furthermore, we observed increased plasma SCAC, which can penetrate the mitochondrial membrane without a carnitine shuttle and serve as additional fuel for FAO and the TCA cycle^4,6–8,42^. This shift towards fatty acid metabolism might also explain that oxygen consumption was increased, as both SCFA and MCFA can effectuate increased oxygen consumption and supply of NADH or FADH2 to the mitochondria^9–11,42^. Additionally, mitochondrial proteomic-analysis showed a tendency towards increased expression of proteins involved in FAO and substrate transport, as well as proteins connecting glycolysis, TCA cycle, FAO, and the electron transport chain. Altogether our findings suggest that the liver increases metabolic activity and more importantly, that these differences already take place in the brain-dead donor, before subsequent preservation and transplantation-related ischemia-and reperfusion injury which is known to further deplete ATP stores^10,43^.

The adaptive hepatic response is in sharp contrast to the metabolic changes observed in the kidneys. Our data suggest that anaerobic glycolysis is enhanced in concert with ATP depletion, increased levels of oxidative stress and decreased renal perfusion. Since oxygen consumption, mitochondrial oxygen utilization, and oxygen saturation levels were not affected, altered renal perfusion and thereby hypoxia likely underlie the observed effects, as opposed to mitochondrial dysfunction. This is supported by a recent study by our group that shows that several markers of hypoxia signalling pathways are increased following 4 hours of BD^11,12,27^. As decreased ASL signals were observed despite normal blood pressure levels, we propose that microcirculatory changes as seen in acute kidney injury (AKI) and sepsis models, underlie this effect. In these models, initial hypoxic damage leads to microcirculatory changes and subsequently decreased renal perfusion, function, and ATP levels, hours later^4,6–8,13,44,45^. Translating this to the BD setting, initial hypoxia is likely caused by the catecholamine storm, which leads to severe hypertension and decreased renal perfusion immediately after BD onset^14,15,25,46^. This immediate decrease in renal perfusion was not reflected in our ASL data, probably because it could, at the earliest, be measured approximately 10 minutes after BD onset, when the effects of the catecholamine storm on renal perfusion have already subsided^16,47^. In AKI, secondary microcirculatory failure is mediated by increased superoxide levels and results in increased lactate/pyruvate ratios, suggesting increased anaerobic metabolism^16,17,48^. Since superoxide levels increase^7,18^ and renal perfusion decreases as BD progresses, these changes could mediate the anaerobic effects we observed as BD progressed. In line with this, increased LDH mRNA expression and plasma levels could be explained by higher lactate-to-bicarbonate and lower NAD+/NADH ratios as a consequence of hypoxic changes^19,49^. Surprisingly, whereas AKI and IRI result in increased gluconeogenic activity in the kidney^20,50,51^, renal gluconeogenesis-related *Pck-1* expression was decreased following BD. Even though the mechanism behind this change is unclear, the protein expression of another rate-limiting gluconeogenic enzyme, glucose-6-phosphate 1-dehydrogenase, is also decreased following BD^21,27^, suggesting that the gluconeogenic capacity of the kidney is impaired.

Several studies suggest that the changes we observed are of clinical importance. Firstly, ASL-related changes in perfusion correlate positively with plasma biomarkers in renal allograft recipients during acute rejection^22,23,52,53^. Furthermore, the occurrence of DGF following renal transplantation is preceded by failure to recover aerobic metabolism or improve ATP levels^21,35^. Additionally, we observed not only metabolic anaerobic changes but also increased oxidative stress markers including MDA, which correlate to both DGF and one-year graft function following transplantation^24,54,55^. Altogether, our results suggest that BD impairs renal perfusion and that hypoxic changes are likely responsible for the metabolic changes observed in the brain-dead donor.

Our results highlight the need for an organ-specific approach to facilitate optimal function of the liver and kidneys following BD. Even though ethical and logistical constraints make treatment of brain-dead donors a challenging endeavour, our data suggests that optimisation of donor homeostasis should include haemodynamic stabilisation and metabolic support. Whether in the donor or during machine perfused organ preservation, we suggest that treatment of the liver should include nutrient supplementation to restore energy supplies, for example by supplying carbohydrate metabolites. In brain-dead pigs, glucose infusion efficiently increased hepatic glycogen content and, concomitantly with insulin administration, increased glucose uptake in other tissues as well, thereby decreasing the hepatic energy demand^25,56^. On the other hand, kidney function should be ameliorated by improvement of renal perfusion while supporting cellular detoxification, reverting back to aerobic metabolism and replenishing energy stores. This could encompass haemodynamic stabilisation of donors with dopamine, which reduced levels of DGF following kidney transplantation^26,57^. Other strategies might behold improving renal (micro)circulation by administering vascular dilators or reducing oxidative stress with anti-oxidant treatments, the latter being of interest as oxidative stress markers in brain-dead donors correlate with DGF^27,55^. Furthermore, the implementation of machine perfusion lends itself well to target the liver and kidney individually, and to monitor their metabolic and energetic status as well as resistance to perfusion to evaluate graft quality prior to transplantation.

We acknowledge that several limitations apply to our study. Due to the experimental setup, sham animals were exposed to a longer anaesthetic duration than brain-dead animals, which introduced anaesthetic duration as a possible confounder. However, studies on the effects of sevoflurane administration in mice have shown that sevoflurane does not alter histopathology or function of the liver and kidneys^8,28,58,59^, suggesting that possible short-term effects of this anaesthetic are negligible. Furthermore, we acknowledge that changes in mRNA expression do not take (post)-translational modifications into account, hence these results should be interpreted with caution. Finally, we wish to point out that due to multiple testing we have increased the probability of type I statistical errors, particularly in our proteomics analysis. However, we have chosen to accept this risk as these statistical tests were performed for secondary outcome parameters, which were used to explore patterns that were in support of our primary outcomes parameters (i.e. MRI and mitochondrial respiration).

In conclusion, we show that BD pathophysiology influences systemic metabolic processes, along with organ-specific metabolic changes with noticeable differences between the liver and kidneys. The liver responds to increased metabolic demands by enhancing aerobic metabolism in normally functioning mitochondria, whilst facilitating the use of alternative energy sources. In contrast, the kidneys shut down metabolically and suffer from oxidative stress, shifting towards anaerobic energy production while renal perfusion decreases. An organ-specific, dual approach focusing on metabolic changes and graft perfusion should be part of novel strategies to assess and treat organ grafts in the brain-dead donor or during preservation, and could be the key to improving transplantation outcomes.

## Methods

### Brain death model

Sixteen male, adult Fisher F344 (Harlan, UK) rats (250–300 g) were randomly assigned to the BD (n = 8) or sham-operated (sham) group (n = 8). The experimental protocol was approved by the Danish Animal Experimentation Inspectorate, under The Ministry of Food, Agriculture, and Fisheries (Approval no. 2014-15-2934-01007). All animals received care in compliance with the National Institutes of Health Guide for the Care and Use of Laboratory Animals and the European Convention for the Protection of Vertebrate Animals Used for Experimental and Other Scientific Purposes. Rats were kept in cages in a 12:12 hour light-dark cycle, with a temperature of 21 °C ± 2 °C, and humidity levels of 55% ± 5%. Animals had *ad libitum* access to a standard rodent diet (Altromin, Lage, Germany) and tap water.

The BD model used was previously described by Kolkert *et al*.^28,30,31^, with the following adaptations to ensure MRI compatibility. Animals were anaesthetised using sevoflurane with and FIO_2_ of 100%, intubated via a tracheostomy, and ventilated (MR-compatible Small Animal Ventilator. SA Instrument, Inc. NY. USA.) with the following ventilation parameters: tidal volume of 7 ml/kg of body weight (kg) per stroke, positive end expiratory pressure of 3 cm of H_2_O with an initial respiratory rate of 120 per min, and corrected based on end-tidal CO_2_ (ETCO_2_). Continuous MAP monitoring and volume replacement was performed via cannulas that were inserted in the femoral artery and vein, respectively. Besides a frontolateral hole, drilled for the epidural placement of a no. 4 Fogarty catheter used to induce BD (Edwards Lifesciences Co, Irvine, CA), a second hole was drilled contralaterally for ICP monitoring with a 24G cannula. BD was induced by inflation of the Fogarty catheter; this inflation was ended once the MAP rose above 80 mmHg. BD was confirmed when the ICP superseded the MAP. After 30 mins of BD, the FiO_2_ was lowered to 50%. Possible reasons for exclusion of animals from the study was the inability to confirm BD or maintain a normotensive MAP. Rocuronium (0.1 mg/ml at 0.3 ml/h) was administered to avoid movements during MR scanning. Sham animals underwent an identical surgical procedure, without insertion of the Fogarty catheter, while anaesthesia lasted the entire 4 hrs of experimental time.

After 4 hrs, the experiment was terminated as previously described^28,32^ and the liver, kidneys, plasma and urine were harvested. Tissue from the liver and one kidney were used for mitochondrial isolation. Additional tissue, plasma and urine were stored.

### Plasma and urine injury markers, blood gas analysis, and metabolites

Plasma levels of AST, ALT, creatinine, urea, LDH, glucose, and lactate, and urine creatinine were determined at the clinical chemistry laboratory of the University Medical Centre Groningen according to standard procedures. Results from one sham animal resulted in supraphysiological values of plasma markers, which was confirmed statistically with an outlier test. As a result, this animal was removed from the analyses. Blood gas analyses were performed immediately after aortic puncturing using ABL725 analysers (Radiometer Medical Aps, Brønshøj, Copenhagen, Denmark) to determine the pH, partial pressure of oxygen, partial pressure of carbon-dioxide, haemoglobin and SaO_2_. Samples containing blood clots were excluded from analyses.

Acylcarnitine analysis was performed using a method previously described^33,34,60^. Supernatant acylcarnitine concentrations were measured with an API 3000 LC-MS/MS, equipped with a Turbo ion spray source (Applied Biosystems/MDS Sciex, Ontario, Canada).

### Glucose metabolism, glycogen storage, and renal injury marker KIM-1

#### Periodic acid–Schiff staining

To determine glycogen tissue concentrations, a PAS staining was performed in paraffin embedded tissue samples. Next, all slices were scanned and ten pictures per slice at 4× (in the case of the liver) or 20× (in the case of the kidney) magnification used to estimate the positive PAS area with ImageJ script^35,61^. The final value per slice was recorded as the mean of positive areas.

#### RNA isolation, cDNA synthesis, and Real-Time quantitative PCR

From whole liver and kidney sections, we isolated total RNA using TRIzol (Life Technologies, Gaithersburg, MD), with a method previously described^8,36,37^. Amplification of several genes fragments was done with the primer sets outlined in Table 1. Pooled cDNA from brain-dead rats was used as an internal reference. Gene expression was normalised with the mean of β-actin mRNA content. Real-Time PCR was carried out according to standard procedures as previously described^11^. Results were expressed as 2^−ΔΔCT^ (CT - Threshold Cycle).

**Table 1:** Primer sequences used for Real-Time PCR.

**Gene**	**Primers**	**Amplicon size (bp)**
*Ho-1*	5′-CTCGCATGAACACTCTGGAGAT-3′ 5′-GCAGGAAGGCGGTCTTAGC-3′	74
*Kim-1*	5′-AGAGAGAGCAGGACACAGGCTTT-3′ 5′-ACCCGTGGTAGTCCCAAACA-3′	75
*Ldha*	5′-AATATTACGTGAAATGTAAGATCTGCATATG-3′ 5′-TTTTCCTTGGCATGACACTTGAG-3′	70
*Pc*	5′-ATCTCTTGCCAAATAAGGGTCTGC-3′ 5′-CAGAGGTAGAACCCCTCTCCCA-3′	88
*Pck-1*	5′-TGTTCTCCGAAGTTCGCATCT-3′ 5′-CTGCTACAGCTAACGTGAAGAACTG-3′	91
*Pfk-1*	5′-GCATAGACAAGGGTTTCTGAGCTTA-3′ 5′-AGCACTGGGAGGGAGAGAGAGT-3′	74
*Pk*	5′-TGGCAGTGTGCAAGGACCA-3′ 5′-CTTTATTATTCATTCCTCTGTCCTCTCC-3′	81

### Mitochondrial respiration

#### Mitochondrial isolation

After 4 hrs of BD, organs were removed and placed into ice-cold 0.9% KCl solution. A differential centrifugation procedure^38,62^ was used to isolate either mitochondria from 1.5 g of liver tissue or a whole kidney. The total volume of working reagent required was determined by calculation of the protein concentration in the mitochondrial suspension with a BCA protein assay kit (Pierce, Thermo Fisher Scientific Inc., Rockford, IL, USA).

#### High resolution respirometry

The rates of oxygen consumption in isolated mitochondria (0.4 mg/ml of mitochondrial protein) were measured at 37 °C using a two-channel high-resolution Oroboros oxygraph-2k (Oroboros, Innsbruck, Austria). The assay medium contained: EGTA (0.5 mM), MgCl_2_ (3 mM), KH_2_PO_4_ (10 mM), Lactobionic acid (60 mM), Taurine (20 mM), HEPES (20 mM), D-Sucrose (110 mM), and bovine serum albumin (BSA, 1 mg/ml, pH 7.2). The substrates for oxidation were: (i) pyruvate (5 mM) + malate (2 mM): TCA cycle function; (ii) succinate (5 mM) + rotenone (1 μM): complex II-dependent respiration; (iii) glutamate (5 mM) + malate (5 mM: glutamate transaminase and TCA cycle function; and (iv) palmitoyl-CoAa (25 μM) + L-carnitine (2 mM) + malate (2 mM): FAO. To reach maximal ADP-stimulated oxygen consumption (state 3), hexokinase (4.8 U/ml), glucose (12.5 mM), and ADP (1 mM) were added. Resting state oxygen consumption rate (state 4) was measured after we blocked ADP phosphorylation with carboxyatractyloside (1.25 μM). The oxygen consumption rate in the uncoupled state (state U) was determined after addition of carbonyl cyanide-4-(trifluoromethoxy) phenylhydrazone (FCCP, 2 μM). The respiratory control ratio (RCR) was calculated by dividing oxygen consumption rate in state 3 by that of state 4. Data acquisition (4 Hz sampling frequency) and analysis were performed using DatLab software version 5 (Oroboros, Innsbruck, Austria).

### MRI assessment of oxygen consumption (BOLD) and perfusion (ASL)

Animals were placed in a MRI compatible animal-bed (Rapid Biomedical, Würzburg, Germany) and the following parameters controlled: rectal temperature, blood and intracranial pressure, ETCO_2_, and pulse oximetry. MRI data was collected with an Agilent 9.4 T preclinical MRI system (Agilent Technologies, Yarnton, UK), containing a 72-mm quadrature 1 H transmit/receive volume coil (Rapid Biomedical, Würzburg, Germany).

A high-resolution coronal spin-echo sequence was employed for anatomical description, acquired using the following sequence parameters: matrix 256 × 192, field of view (FOV) of 107 × 90 mm2, repetition time (TR) of 3000 ms, echo time (TE) of 22.8 ms, and 1 mm thickness.

BOLD MRI relies on differences in oxygenated and deoxygenated haemoglobin concentrations in blood vessels and surrounding tissue. These differences cause contrast in MRI influencing the so-called spin-spin relaxation rate (R2*), allowing R2* values to be correlated to oxygen consumption (when external factors such as regional perfusion are accounted for) and inversely correlated to oxygen availability of a specific region of interest (i.e. low R2* values indicate low oxygen consumption yet high oxygenation)^39,63,64^. R2* can be calculated using an oxygenation-dependent sequence (T2*-weighted) and obtained using an axial 1H-multi-echo gradient-echo sequence, covering the entire abdomen with 32 slices using the following sequence parameters: matrix of 128 × 128, FOV of 80 × 80 mm2, flip angle of 90°, TR of 800 ms, TE of 2, 4, 6, 8, 10, 12, 14, and 16 ms, number of transients of 2, and 2 mm thickness. R2* levels calculated at baseline (time 0 h) served as an internal control to calculate relative changes in oxygenation, a measure superior to absolute values^40,64^.

ASL MRI relies on a difference in the T1-weighted signal of inflowing blood compared to that of the tissue of interest, allowing estimation of relative changes in tissue perfusion. For T1-measurements a single-slice segmented Look–Locker sequence with a gradient-echo readout was used to acquire T1-weighted data. The following sequence parameters were used: matrix of 128 × 128, FOV of 80 × 80 mm2, flip angle of 8°, TR of 3 ms, TE of 2 ms, inversion times (TI) of 150, 250, 400, 600, 900, 1200, 2500, 4000 ms, and 2 mm thickness.

VnmrJ (Agilent Technologies) was used for image reconstruction and volumetric analysis. Manually drawn regions of interest were encompassed around the liver and kidneys. Quantitative T2* maps were calculated from pixel-by-pixel analysis using a nonlinear least-squares fit to the logarithmic magnitude vs. TE. Quantitative T1 maps were calculated from a three-parameter fit applied to the inversion recovery Look-Locker sequence, using the mathematical approach given by Ramasawmy *et al*.^16,65^.

### Mitochondrial proteomics, complex I and ATP synthase activity, and tissue ATP levels

#### Targeted, quantitative mitochondrial proteomics

In isolated mitochondria, we quantified a selection of mitochondrial proteins involved in the substrate transport, FAO and TCA cycle, using isotopically-labelled standards (^13^C-labeled lysines and arginines). These were derived from synthetic protein concatamers (QconCAT) (PolyQuant GmbH, Bad Abbach, Germany), using a method previously described^66^.

#### Hepatic and renal ATP concentrations

Frozen liver and kidney tissue was cut into 20 mm slices; 650 mg of these slices were used to determine ATP content according to standard procedures^67^.

#### Complex I and ATP synthase enzyme activity measurements

Mitochondria were isolated as previously described^62^, then diluted in PBS, lysed by sonication, and centrifuged at 600 g for 10 min at 4 °C. Protein concentration was determined in the supernatant using a BCA protein assay kit (Pierce, Thermo Fisher Scientific Inc., Rockford, IL, USA).

Activity of complex I was monitored spectrophotometrically at 600 nm and 37 °C, as previously described^68^. Rotenone-sensitive complex I activity was calculated using the molar extinction coefficient of DCIP, equal to 21000 M^−1^cm^−1^ and expressed as nmol/min/mg protein^68^. The activity of ATP synthase was measured spectrophotometrically at 340 nm and 37 °C, as previously described^64^. Oligomycin-sensitive ATP synthase activity was calculated using the molar extinction coefficient of NADH, equal to 6220 M^−1^cm^−1^ and expressed as nmol/min/mg mitochondrial protein^69^.

### Oxidative stress markers

#### Determination of oxidative damage through quantification of lipid peroxidation

Lipid peroxidation product MDA was quantified in liver and kidney homogenates (20 µl) by measuring the formation of thiobarbituric acid reactive substances with a method previously described^7^.

#### Gene expression of protective protein Heme oxygenase-1

Gene expression of *Ho-1* was determined with Real-time PCR as described previously^8^ with the primer set outlined in Table 1.

### Statistical Analyses

For *R2 BOLD and T1 data, we estimated that we would need a total of 8 animals per group to detect a clinically significant difference with an α of 0.05, power of 80%, using a two-tailed test. Descriptive statistics were done to confirm that the data met the assumption of equal distributions of residuals. A linear mixed model was used with repeated measures over time to analyse the impact of the treatment (BD or sham) on BOLD and ASL in the liver and kidney, with fixed effects of time, treatment group, and the interaction of treatment and time (IBM SPSS Statistics 23). This model was chosen because it takes the dependency of the measurements across time into consideration, and prevented list-wise deletion caused by missing data points. The model selection for covariance parameters was chosen based on the best fit according to the Bayesian Information Criterion. When comparing two independent groups at a single time point, the non-parametric Mann-Whitney test was used to identify significant differences between the groups (n = 8 in each group) with Prism 6.0 (GraphPad Software Inc, CA, USA). To confirm abnormal results, a boxplot was performed to identify extreme outliers and considered significant when they scored >3× IQR compared to the other values with IBM SPSS Statistics 23. All statistical tests were 2-tailed and *p* < 0.05 was regarded as significant. Results are presented as mean ± SD (standard deviation).

### Availability of data and materials

All data generated or analysed during this study are included in this published article (and its supplementary information files).

## Electronic supplementary material

Supplementary figures
