# Supplementary material for: Organ-specific responses during brain death: increased aerobic metabolism in the liver and anaerobic metabolism with decreased perfusion in the kidneys
Source: Sci Rep. 2018 Mar 13;8:4405. doi: 10.1038/s41598-018-22689-9 (PMC5849719; doi:10.1038/s41598-018-22689-9)
Supplement: Supplementary file 1 — Supplementary figures [file 41598_2018_22689_MOESM1_ESM.doc]

**Organ-specific responses during brain death: increased aerobic metabolism in the liver and anaerobic metabolism with decreased perfusion in the kidneys.**

Van Erp AC.^1*^, Rebolledo R.^1,2*^, Hoeksma D.^1^, Jespersen NR.^3^, Ottens PJ.^1^, Nørregaard R.^4^, Pedersen M.^5^, Laustsen C.^5^, Burgerhof JGM.^6^, Wolters JC.^7,8^, Ciapaite J.^8,9^, Bøtker HE.^3^, Leuvenink HGD.^1^ and Jespersen B. ^4,10^

**Supplementary figures**

**
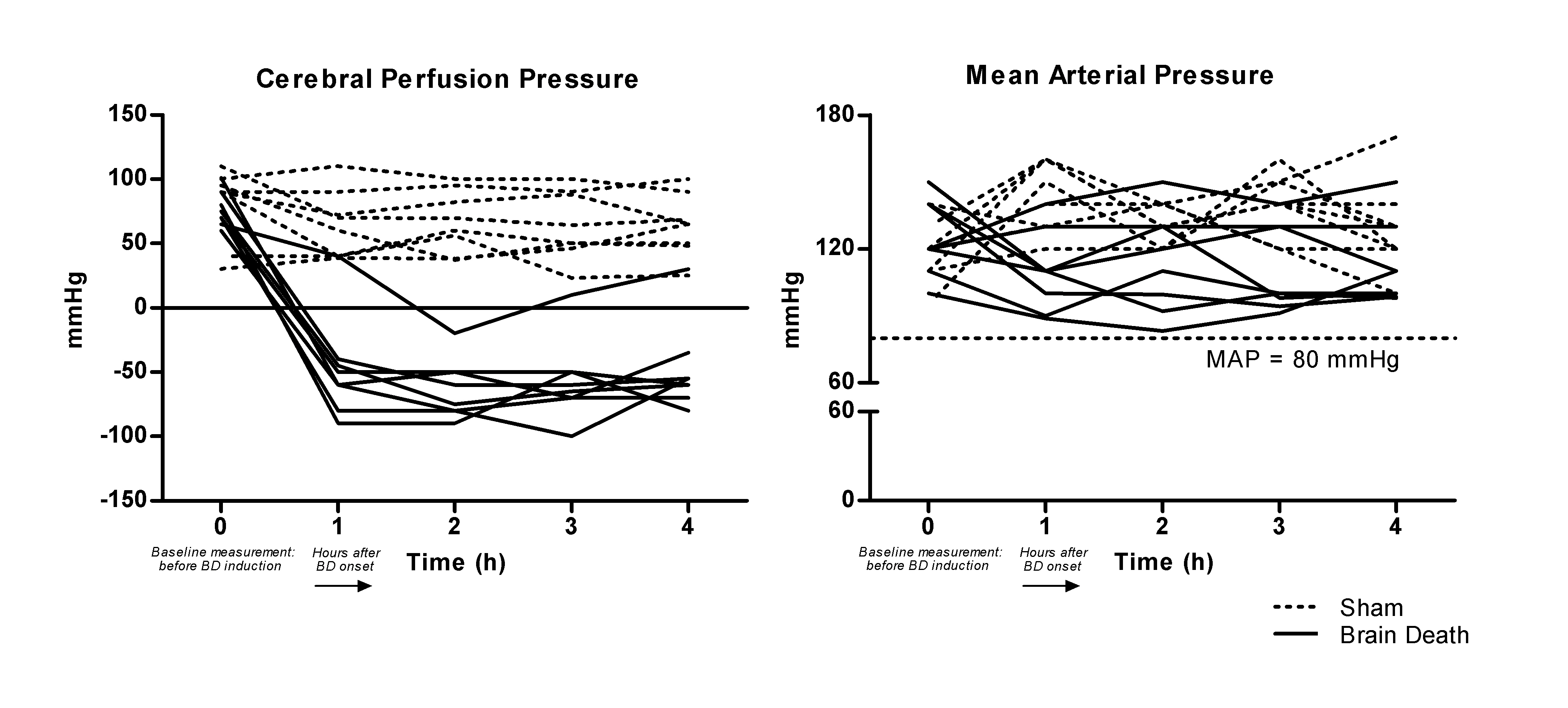
**

**Figure S1. Brain death reduced cerebral perfusion pressure and mean arterial pressure**.

Time point “0” represents the baseline measurement before BD induction. Subsequent time points refer to hours after confirmation of BD. **A)** Cerebral perfusion pressure profile, measured by subtracting intracranial pressure from the mean arterial pressure (MAP). **B)** MAP profile. Results are presented as mean ± SD, n = 8 per group.


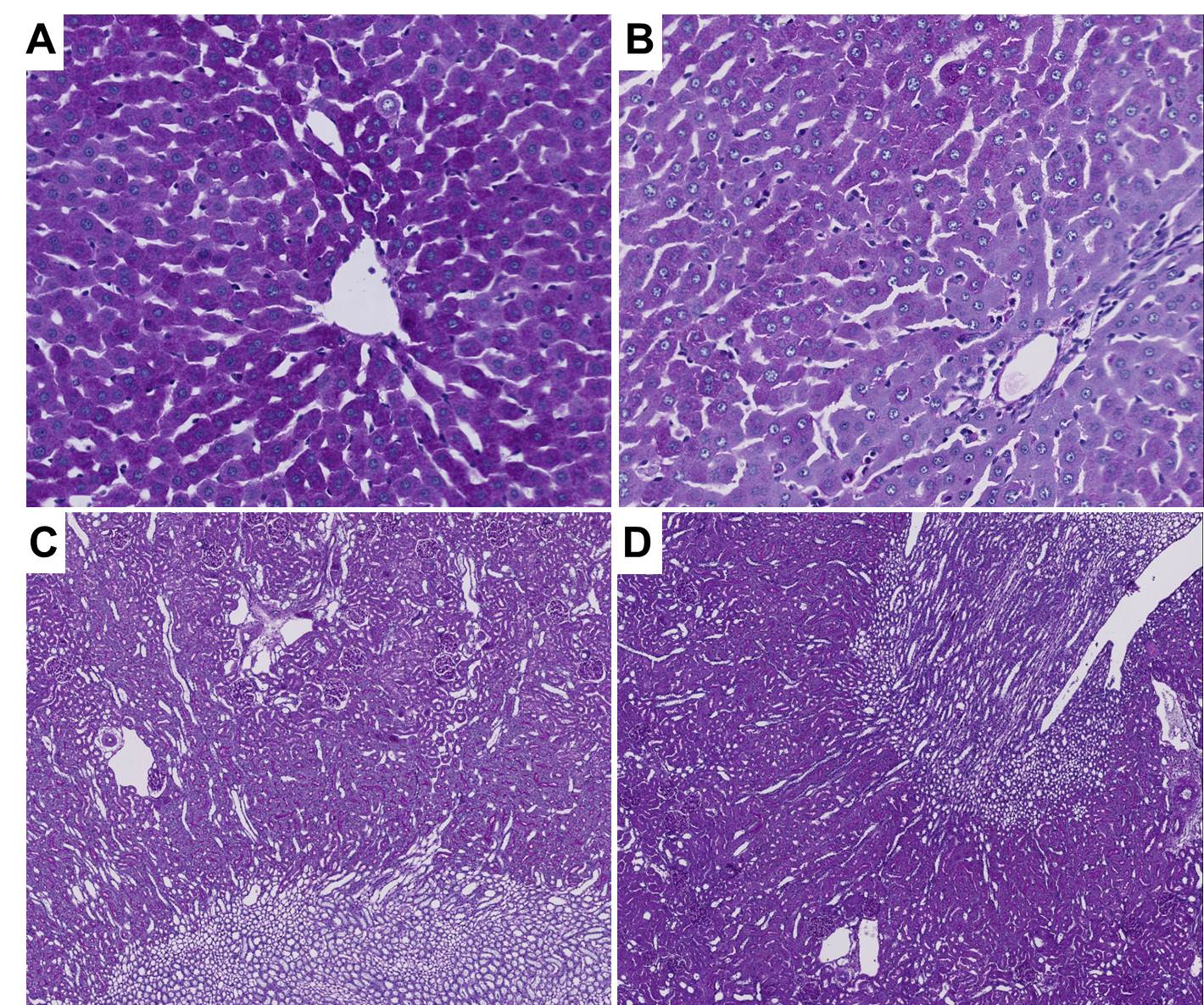


**Figure S2. Liver glycogen levels estimated with Periodic Acid–Schiff (PAS) staining.** Immunohistochemistry staining in the **A, B)** liver of a sham (left) and brain-dead (right) animal at 20 x original magnification, and **C, D)** kidney of a sham (left) and brain-dead (right) animal at 4 x original magnification. The sections are representative of 8 independent rats per group.


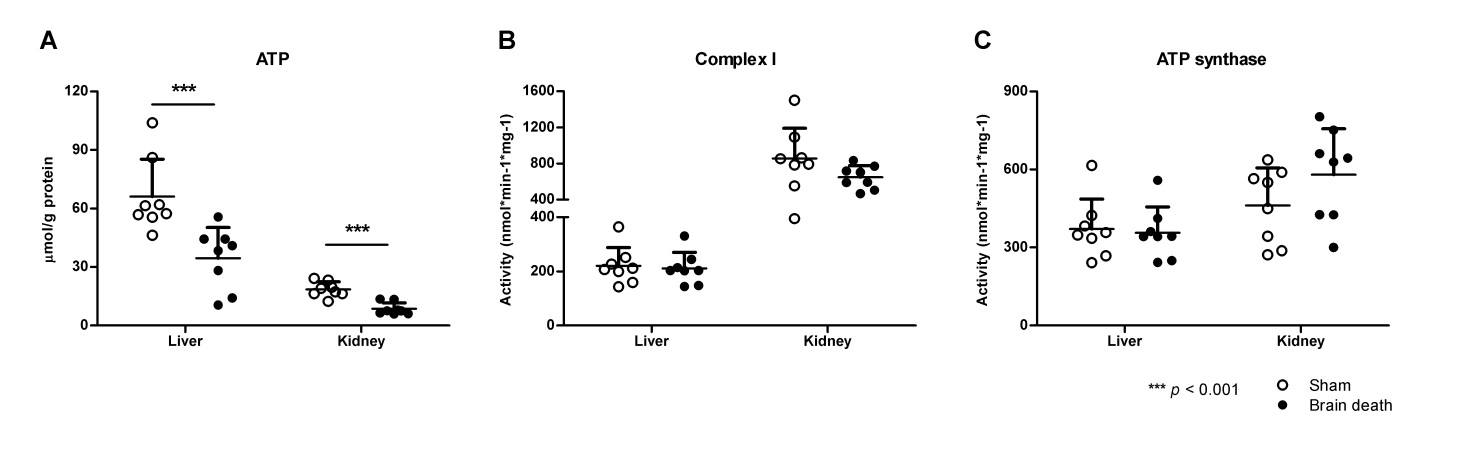


**Figure S3. Decreased ATP levels, yet unaltered activities of mitochondrial complex I and ATP synthase in the liver and kidneys following brain death.**
**A)** ATP content in liver and kidney tissue; **B)** Complex I and **C)** ATP synthase activity in isolated renal and hepatic mitochondria. Results are presented as mean ± SD, n = 8 per group (*** *p* < 0.001).


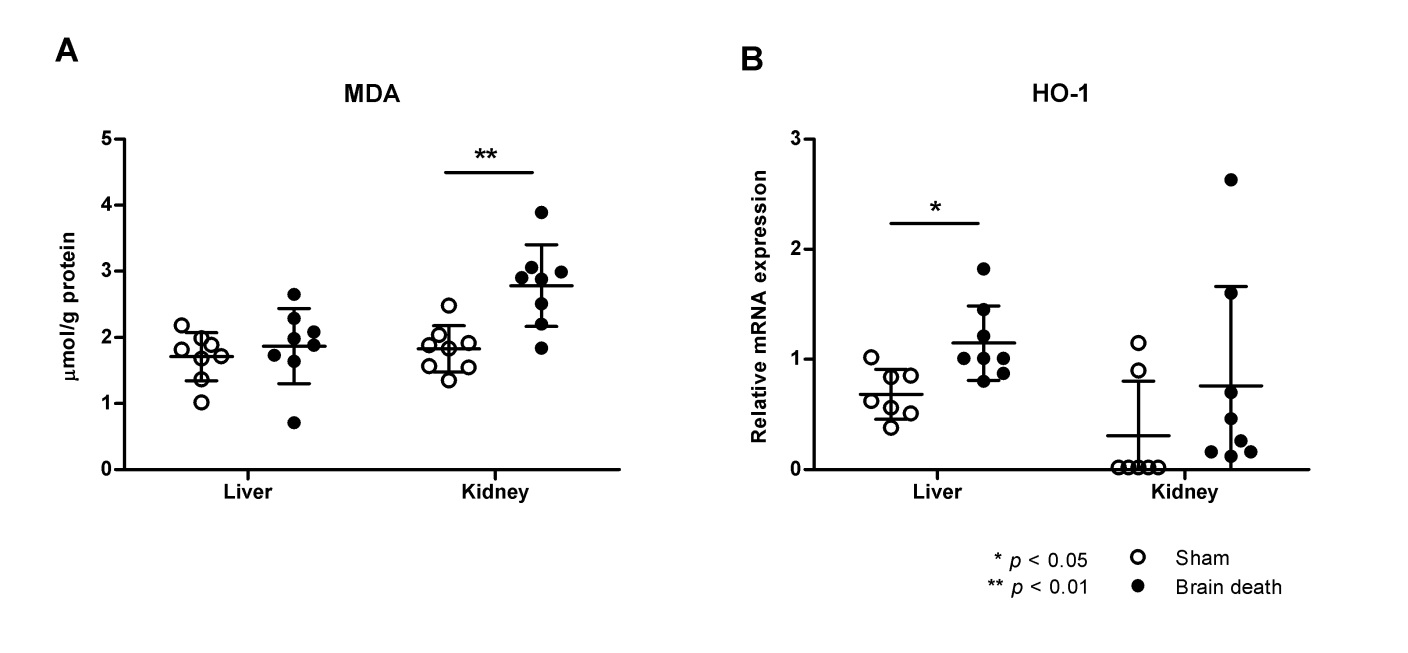


**Figure S4. Increased oxidative stress in the kidney, but not the liver, following brain death.**

**A)** Malondialdehyde (MDA) and **B)** Relative gene expression of protective protein Heme Oxygenase 1 (*Ho-1*) in liver and kidney tissue of brain-dead and sham animals. Results are presented as mean ± SD, n = 8 per group (* *p* < 0.05, ** *p* < 0.01).
